# The cost of cuts

**DOI:** 10.1038/s43856-021-00026-y

**Published:** 2021-08-25

**Authors:** Ben Abbott

**Affiliations:** Communications Medicine, https://www.nature.com/commsmed/

## Abstract

Improvements in life expectancy have stalled over the last ten years in England, prompting research into the underlying causes. A recent study in *The Lancet Public Health* examines the relationship between cuts to local government funding and mortality in England.

**Figure:**
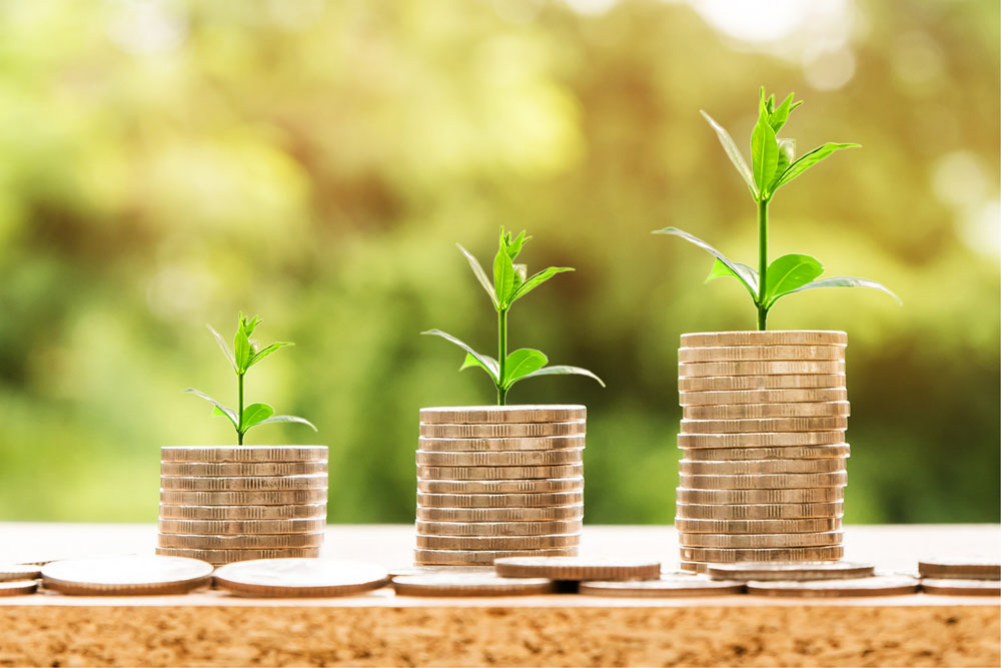
Pixabay

Despite fluctuations in yearly mortality rates, the last 50 years have generally seen a steady increase in life expectancy in high-income countries. Life expectancy has plateaued in England in the last decade, however, with cuts in government spending a potential contributing factor.

The UK government centrally funds healthcare but also allocates funding to local authorities to spend on housing, social care, public health, environmental services and other public services, all of which may directly or indirectly influence health outcomes. Funding to local governments was cut drastically as part of the austerity measures implemented in the wake of the 2008 financial crisis.

A recent study by Alexiou and colleagues in *The Lancet Public Health* explores the potential contribution of cuts in government funding to England’s stalled life expectancy^1^. The authors conduct a longitudinal ecological study, collecting data on central government funds received by 147 local authorities in England from 2013 to 2017 and associating funding levels with male and female life expectancy at birth during the same period.

The authors found that central government funding to local authorities decreased on average by £168 per person during the study period. Local authorities with a greater reduction in funding showed a slower growth in life expectancy, with some even showing a decline over time. Each annual £100 per-person reduction in funding was associated with an estimated 1.3-month and 1.2-month reduction in life expectancy at birth in males and females, respectively. Reductions in funding were greater in more deprived areas and these areas also had the worst changes in life expectancy.

Although the authors adjust for some confounding factors such as unemployment rate, they note that other confounders may remain. It is also important to recognise that this study does not prove causation, and further work would be needed to establish causal mechanisms between government cuts and reduced life expectancy.

With these limitations in mind, the study highlights the potential disproportionate impact of government spending policies on different areas and the likelihood that funding cuts reinforce existing health inequalities. Addressing such inequalities might help to ensure all of us can live equally long lives.
